# From Haiti to the Amazon: Public Health Issues Related to the Recent Immigration of Haitians to Brazil

**DOI:** 10.1371/journal.pntd.0002685

**Published:** 2014-05-08

**Authors:** Tom Rawlinson, André Machado Siqueira, Gilberto Fontes, Renata Paula Lima Beltrão, Wuelton Marcelo Monteiro, Marilaine Martins, Edson Fidelis Silva-Júnior, Maria Paula Gomes Mourão, Bernardino Albuquerque, Maria das Graças Costa Alecrim, Marcus Vinícius Guimarães Lacerda

**Affiliations:** 1 Western General Hospital, Edinburgh, United Kingdom; 2 Fundação de Medicina Tropical Dr. Heitor Vieira Dourado, Manaus, Amazonas, Brazil; 3 Universidade do Estado do Amazonas, Programa de Pós-Graduação em Medicina Tropical, Manaus, Amazonas, Brazil; 4 Universidade Federal de São João Del Rei, Campus Centro Oeste, Divinópolis, Minas Gerais, Brazil; 5 Fundação de Vigilância em Saúde, Manaus, Amazonas, Brazil; Centers for Disease Control and Prevention, United States of America

## Haitian Migration to Brazil

**Figure 1:**
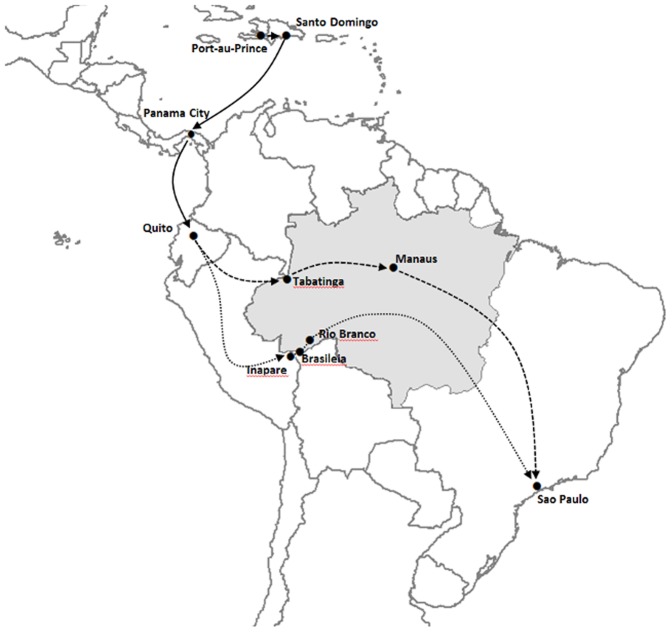
Migration routes for Haitians to Brazil. Usual routes begin with flights to Panama City from Santo Domingo and from there to Quito. Entry into Brazil is either through Tabatinga and then on to Manaus (dashed line) or to Brasileia and Rio Branco (dotted line). Although most Haitians remain in Manaus or Rio Branco, some have since moved to Sao Paulo, Brazil's largest city. The grey area represents the Brazilian Amazon region.

In late 2010, Haitian immigrants began to arrive at remote river border crossings in the western Brazilian Amazon. Attracted by the prospect of work in Brazil's burgeoning economy, thousands of Haitians paid large sums to people traffickers, known as “coyotes,” to arrange their journey to Brazil. They entered Brazil through the border towns of Tabatinga (Amazonas state) and Brasileia (Acre state) (Figure 1) [1], [2]. Their journeys from Haiti were complex and involved travel by air, road, river boat, and on foot. Between four and six thousand Haitians have arrived in Brazil since 2010 [1]. Most have made their way to Manaus, a city of 1.8 million inhabitants in the western Amazon. Manaus itself presents considerable challenges for infectious disease control because of its dynamic mix of urban and forest environments and its fringe of shanty towns.

Brazil's national health system—Sistema Único de Saúde (SUS)—is based on the principles of universal access, decentralization, and social participation [3], [4]. One of its major achievements to date has been to reduce the burden of several infectious diseases by successful control programmes and high immunization coverage rates [5].

Haiti's public health situation is in stark contrast to that of Brazil. Since the 2010 earthquake, the Haitian health infrastructure has been massively overstretched and incapable of handling the population's many needs. There has been heavy reliance on the services of the United Nations (UN) and international nongovernmental organizations.

A survey conducted in 2009 showed vaccine coverage of only 40% among children in Haiti [6].

In addition to high rates of infectious diseases such as malaria, tuberculosis, and lymphatic filariasis, the Haitian population now faces a devastating cholera epidemic [7], [8].

Brazil has stated its humanitarian commitment to the welfare of the Haitian immigrants and in fulfilling this faces two main health-related challenges:

1. To provide adequate medical care to the newly arrived Haitians. This is complicated by linguistic barriers, as few Brazilians in the region are fluent in French and almost none are fluent in Creole.
2. To identify infectious diseases that might be reintroduced via immigration from epidemic and endemic countries and apply the proper measures to minimise the risk of this.

## Methods

In this discussion paper, we focus on lymphatic filariasis and cholera. These diseases are in an advanced phase of control in Brazil, the former in a near-elimination stage and the latter with no transmission reported since the 1990s. However, in the case of both of these diseases, the Brazilian Amazon remains vulnerable to reintroduction. Articles relevant to assessing the risk of reintroduction of these diseases were identified through PubMed searches.

The authors met with members of the Haitian immigrant community to understand their journey and gain insights into their state of health and living conditions.

International experts in the epidemiology of cholera and lymphatic filariasis were consulted and kindly offered their opinions on the particularities of this context.

## Lymphatic Filariasis (LF)

This parasitic disease caused by the nematode worm *Wuchereria bancrofti* is estimated to affect 108 million individuals worldwide, with 1,390,000,000 people living in regions of transmission [9], [10]. The spectrum of clinical manifestations ranges from asymptomatic infection to chronic and debilitating complications, most notably lymphoedema and elephantiasis [10], [11]. The economic losses and social stigma caused by the physical deformities of the chronic phase affect around 40 million people and make lymphatic filariasis (LF) the second largest cause of disability worldwide [12].

In the Americas, there is still active transmission in Haiti, the Dominican Republic, Guyana, and Brazil [10], [13]. In both Haiti and Brazil, the same mosquito vector, *Culex quinquefasciatus*, is responsible for transmission. From 11 cities endemic for LF in the 1950s, Manaus amongst them [14], transmission in Brazil is now restricted to just one region in the northeast—the metropolitan area of Recife [13]. The primary vector, *C. quinquefasciatus*, is present in high densities and is the most frequent indoor mosquito throughout the year in the city of Manaus [15].This high vector density underlies the potential for the transmission of lymphatic filariasis to reemerge.

There are some reports in the literature of LF accompanying migration; in Sri Lanka, infected migrants have engendered LF in areas where the disease was previously unknown [16], and similarly, in metropolitan Recife in Brazil, cases of LF have appeared in previously unaffected areas [17]. A study in Haiti demonstrated a significant increased risk of being a positive individual when residing within 20 meters of a case [18]. Different studies have reached distinct conclusions regarding the potential risk of LF reintroduction, and as the literature is scarce and the risk hard to quantify, continuous surveillance is essential [19], [20].

The main factors affecting the transmission of LF are the local density of microfilariae carriers, poor water, and sanitation conditions favouring rapid population growth of the insect vectors and suitable environmental conditions (temperature >25°C and relative humidity >80%), which promote development of filarial larvae in mosquitoes [21].

The majority of filariasis patients are asymptomatic despite detectable microfilariae in their peripheral blood. These asymptomatic carriers act as a reservoir of infection, with a strong correlation between the prevalence of microfilaraemia and risk of annual transmission potential, and require epidemiological attention to successfully interrupt transmission of the parasite [22]. Thus, it is fundamental that efforts are made in identifying these asymptomatic carriers, as adult filarial worms can survive in the human host for up to ten years, releasing microfilariae found in the bloodstream of the infected person. These findings establish the need for sustained long-term strategies with mass screening and treatment of asymptomatic carriers in order to achieve elimination [23], [24].

In Brazil, intervention strategies initially focused primarily on active case detection by microscopic blood analysis and selective treatment with diethylcarbamazine (DEC). Since 2003, Mass Drug Administration (MDA) with DEC alone or in combination with ivermectin has been undertaken in areas of transmission. Follow-up studies after MDA have indicated interrruption of filarial transmissiton and shown that microfilaremia rates have declined to very low levels.

Estimates for *W. bancrofti* infection in Haiti are around 10%, and the entire population of the country is considered at risk of infection [25]. It is likely that programmes of mass treatment established in 2001 will have had some impact in reducing the prevalence of infection although there are currently no updated surveys.

Preliminary efforts have already begun in Manaus to investigate rates of microfilaria carriage among the Haitians by active testing through blood smear and rapid test, with treatment being given to positive individuals regardless of symptoms. This is important in that, as well as facilitating the provision of proper care and eradicative treatment to the immigrant group, it may reduce the risk of recontamination of local vector mosquito populations.

## Cholera

In October 2010, Haitian authorities reported an increase in cases of acute diarrhoea. Within days, the National Public Health Laboratory had isolated *Vibrio cholerae* serogroup O1, serotype Ogawa. Over the following month, cholera cases emerged in all ten departments of Haiti, and nearly 1,000 deaths were reported. In the three years since, over 7,436 deaths and more than 604,000 cases have been recorded [26], [27]. The UN expert committee report concluded that the source of the Haitian cholera outbreak was contamination of the Meye Tributary of the Artibonite River with a pathogenic strain of current South Asian type *V. cholerae* by a human source [28], [29]. Whole genome sequencing of the *V. cholerae* strain circulating in Haiti has shown it to be very similar to a strain circulating in a Nepali epidemic just prior to Nepali UN troops being deployed to Haiti [29]. The explosive spread and devastating effect of the epidemic in Haiti has been attributed to a “perfect storm” of factors, including the immunological naivety of the population to cholera, the very poor water and sanitation conditions, the optimal environmental conditions for *V. cholerae* proliferation, and the particular virulence of the South Asian strain of *V. cholerae* [30].

The last confirmed autochthonous case of cholera in Brazil was registered in 2005 [5]. An epidemic in the 1990s caused 161,432 cases and 1,296 deaths, most of them in the poorest (north and northeast) regions of the country [5], [31].

To assess the risk of cholera being reintroduced to Brazil in a context such as this Haitian immigration, there are two main questions to consider: Does Brazil remain susceptible to another cholera epidemic? What is the likelihood of prolonged asymptomatic carriage among the Haitian immigrants?

Despite considerable reduction in poverty in recent years, vast social disparities remain in Brazil. In some major urban centres, sanitary conditions are comparable with those in developed Western countries, whereas many peripheral and rural areas still lack proper sanitation, being more similar in this respect to developing and cholera-endemic countries in Africa and Asia [32]–[35]. Particularly in the northern Amazon region, the recent occurrence of waterborne diarrhoeal outbreaks [36]–[38] demonstrates the precarious sanitary conditions of these areas and alerts us to their ongoing vulnerability to a disease such as cholera. The expansion of the Haitian epidemic to the Dominican Republic and Cuba demonstrates the risk of the disease spreading throughout the region and should engender caution in other vulnerable countries in Central and South America [39], [40].

The generally accepted notion is that prolonged asymptomatic carriage of cholera is rare and poses a minimal risk of transferring the bacteria from place to place over significant time intervals and distances. Evidence of the low risk of transmission from convalescent individuals comes from the study by Pierce et al., in which the authors were able to detect viable vibrios only from duodenal fluid or through induced purging [41]. The lack of identification of positive cases in Brazil despite specific control measures for three years may corroborate the low risk of transmission from convalescent individuals, which is yet to be properly determined.

The few studies looking at this issue are old, small, and mostly based in endemic countries where there may be background immunity and reinfection is likely. The well-known case of Cholera Dolores, described in the Philippines in 1967, was the first documented case of a long-term carrier; cholera vibrio, not thought to be from reinfection, were cultured in her stool for several years. The authors concluded that “this patient, with limited chance of transmission, appears to be harmless in the endemic area where she lives, but the impact of her presence in a suitable environment among a susceptible population with a good chance of transmission might be entirely different” [42]. Indeed, continuous and active surveillance must be maintained, reinforced by the diagnosis in 2011 in Sao Paulo of cholera in a patient who had been in the Dominican Republic [5].

At the beginning of the 1990s' epidemic in Peru, rectal swabs were taken from 5,992 asymptomatic people with no history of diarrhoeal disease, and approximately 3% of these swabs grew vibrio cholerae [43]. This Peruvian population, like the Haitians, was relatively cholera naïve at that time.

A recent paper in Nature reports that “the asymptomatic ratio in cholera is far higher than had been previously supposed” and that “inapparent infections can hold the key to interpreting disease outbreaks” [44].

A study from Nigeria followed 13 cholera convalescent patients with twice weekly stool cultures and rectal swabs and showed that 69% of the convalescents had positive cholera faecal cultures for periods ranging from two weeks to more than seven months [45]. However, as in all endemic-region studies, reinfection rather than long-term shedding could not be discounted.

These studies, whilst giving insights into the rates of asymptomatic infection and the duration of shedding, are not directly applicable to the question of the Haitian immigration to the Amazon.

There do not appear to have been any studies assessing the length of asymptomatic carriage in immunologically naive subjects who have moved to nonendemic settings.

Given that the Haitian epidemic's origin seems to have been via long-distance, long-term asymptomatic carriage of the vibrio [28], this question of asymptomatic carriers shedding viable organisms in stool is of major epidemiological importance and needs further investigation.

In the absence of good evidence to provide reassurance on this issue, a precautionary approach may be reasonable. Treatment with single-dose azithromycin has been shown to be effective at eliminating *V. cholerae* carriage [46], [47].

The UN independent expert panel's final report into the Haitian cholera epidemic recommends the following: “To prevent introduction of cholera into nonendemic countries, UN personnel travelling from endemic areas should either receive a prophylactic dose of appropriate antibiotics before departure or be screened with a sensitive method to confirm absence of asymptomatic carriage of vibrio cholerae or both” [48].

## Conclusions

As Brazil increasingly turns into an attractive destination for economic migration from the Americas, Africa, and Asia, the public health system must prepare itself to properly respond to the public health issues this raises.

With particular reference to the recent movement of Haitians to the Brazilian Amazon and the two diseases considered in this article, the authors wish to propose four main points for consideration and debate:

1. The Haitian immigrants should be offered testing for and, if positive, treatment to eliminate *W. bancrofti* microfilariae carriage;
2. in light of the UN expert panel's recent recommendation of mass screening and/or presumptive antibiotic treatment as a means to prevent introduction of cholera, the authors propose that this be considered in a situation such as this where there is a mass movement of people from an epidemic zone to a cholera-free but vulnerable zone. This is also a unique opportunity to study the extent and implications of long-term asymptomatic carriage of cholera and its role in the international spread of the disease.
3. Due to the peculiar geography of the Amazon and the lack of health personnel in these areas, the close collaboration of civil, military, governmental, and nongovernmental organizations is crucial for adequate infectious disease surveillance and control.
4. Newly arrived immigrant groups are a very vulnerable population, and any public health or research initiatives need to be conducted in a very sensitive and culturally appropriate manner.
